# HIV Malaria Co-Infection Is Associated with Atypical Memory B Cell Expansion and a Reduced Antibody Response to a Broad Array of *Plasmodium falciparum* Antigens in Rwandan Adults

**DOI:** 10.1371/journal.pone.0124412

**Published:** 2015-04-30

**Authors:** Krishanthi S. Subramaniam, Jeff Skinner, Emil Ivan, Eugene Mutimura, Ryung S. Kim, Catherine M. Feintuch, Silvia Portugal, Kathryn Anastos, Peter D. Crompton, Johanna P. Daily

**Affiliations:** 1 Vector Biology Department, Liverpool School of Tropical Medicine, Pembroke Place, Liverpool, United Kingdom; 2 Malaria Infection Biology and Immunity Unit, Laboratory of Immunogenetics, NIAID/NIH, Bethesda, Maryland, United States of America; 3 Department of Biomedical Laboratory Sciences, University of Rwanda, College of Medicine and Health Sciences, Kigali, Rwanda; 4 Regional Alliance for Sustainable Development, Kigali, Rwanda; 5 Department of Epidemiology and Population Health, Albert Einstein College of Medicine, Bronx, New York, United States of America; 6 Department of Microbiology and Immunology, Albert Einstein College of Medicine, Bronx, New York, United States of America; 7 Department of Medicine, Albert Einstein College of Medicine, Bronx, New York, United States of America; University of Massachusetts Medical School, UNITED STATES

## Abstract

HIV infected individuals in malaria endemic areas experience more frequent and severe malaria episodes compared to non HIV infected. This clinical observation has been linked to a deficiency in antibody responses to *Plasmodium falciparum* antigens; however, prior studies have only focused on the antibody response to <0.5% of *P*. *falciparum* proteins. To obtain a broader and less-biased view of the effect of HIV on antibody responses to malaria we compared antibody profiles of HIV positive (HIV+) and negative (HIV-) Rwandan adults with symptomatic malaria using a microarray containing 824 *P*. *falciparum* proteins. We also investigated the cellular basis of the antibody response in the two groups by analyzing B and T cell subsets by flow cytometry. Although HIV malaria co-infected individuals generated antibodies to a large number of *P*. *falciparum* antigens, including potential vaccine candidates, the breadth and magnitude of their response was reduced compared to HIV- individuals. HIV malaria co-infection was also associated with a higher percentage of atypical memory B cells (MBC) (CD19+CD10-CD21-CD27-) compared to malaria infection alone. Among HIV+ individuals the CD4^+^ T cell count and HIV viral load only partially explained variability in the breadth of *P*. *falciparum*-specific antibody responses. Taken together, these data indicate that HIV malaria co-infection is associated with an expansion of atypical MBCs and a diminished antibody response to a diverse array of *P*. *falciparum* antigens, thus offering mechanistic insight into the higher risk of malaria in HIV+ individuals.

## Introduction

Individuals with HIV infection who live in malaria endemic areas experience more frequent and severe malaria episodes, but the immunological basis of this clinical observation remains unclear [1, 2]. Antibodies are known to play a central role in protection against the blood-stage of *Plasmodium falciparum* malaria [3–5], and previous studies suggest that HIV infected individuals mount sub-optimal antibody responses to *P*. *falciparum* infection [6–8]. However, these studies only examined antibody responses to <0.5% of *P*. *falciparum* proteins. Studies indicate that both the breadth (number of antigens recognized by antibodies) and magnitude (level of antibodies) of the antibody response to *P*. *falciparum* antigens are critical for protection from malaria in immunocompetent individuals [9–13]. Whether HIV infection has a generalizable effect on the overall breadth and magnitude of *P*. *falciparum*-specific antibody responses remains an open question.

Interestingly, both HIV and malaria are associated with antibody responses that do not reliably and efficiently protect from disease progression [14, 15]. Moreover, both HIV and malaria have separately been associated with a skewing of the B cell response toward a phenotypically distinct subset of isotype-switched, somatically hypermutated B cells that lack the canonical CD27 marker of classical memory B cells (MBCs) and upregulate several inhibitory receptors [16–21]. It has been speculated that this B cell subset, referred to as exhausted or atypical, is related to the inefficient antibody response to HIV and malaria. Whether HIV malaria co-infection further skews the B cell response toward an exhausted or atypical profile is also unknown.

Here we sought to obtain a broader and less-biased view of the effect of HIV infection on the overall breadth and magnitude of *P*. *falciparum*-specific antibody responses by examining the IgG response to 824 *P*. *falciparum* antigens by protein microarray in HIV positive (HIV+) and HIV negative (HIV-) Rwandan adults with symptomatic *P*. *falciparum* malaria. We also sought to understand the cellular basis of the antibody response to malaria in the context of HIV co-infection by analyzing B cell subsets by flow cytometry in the same individuals.

We observed that HIV+ individuals are capable of generating IgG to a large number of *P*. *falciparum* antigens, including potential vaccine candidates, however, the overall breadth and magnitude of this response was reduced compared to HIV- individuals. We also found that HIV+ individuals with malaria had a higher percentage of atypical MBCs compared to HIV- individuals with malaria. Interestingly, the breadth and magnitude of *P*. *falciparum*-specific antibody response in HIV+ individuals was not fully explained by the CD4^+^ T cell count and HIV viral load, suggesting that other factors play a role in generating robust antibody responses to malaria. Taken together, these data indicate that HIV infection has a generalizable negative impact on the overall breadth and magnitude of *P*. *falciparum*-specific antibody responses, and that HIV malaria co-infection is associated with an expansion of atypical MBCs beyond that induced by malaria alone. These observations provide important insights into the immunological basis of increased risk of malaria in HIV+ individuals.

## Material and Methods

### Study population

We recruited subjects with mild malaria from five health clinics around Kigali, Rwanda during the malaria transmission season from January to April 2011. Rwanda is a hypo-endemic region for malaria [22]. Mild malaria was defined as any level of symptomatic *P*. *falciparum* parasitemia without evidence of vital organ dysfunction [23]. Adult subjects (>18 years) who presented with symptoms consistent with mild malaria and had a positive malaria smear (of any parasitemia) were offered enrollment. The inclusion criteria required a positive confirmatory thick blood smear and a positive malaria rapid diagnostic test (First Response Malaria Antigen Rapid Test, Premier Medical Corporation, Uttar Pradesh, India). All enrolled subjects underwent an HIV test and those with a new HIV diagnosis were offered counseling and evaluation. HIV testing was done using the Abbott Determine Rapid Test Strips for HIV-1/2 (Abbott Laboratories, Princeton, New Jersey) and the Uni-Gold HIV Rapid Test (Trinity Biotech, Ireland). At enrollment each subject underwent a review of symptoms and physical exam and received artemether-lumefantrine for treatment of malaria. Medical records were reviewed in subjects receiving HIV care to obtain the history of opportunistic infections, anti-retroviral (ARV) medications and CD4^+^ T cell counts. Percent parasitemia was calculated from five different microscopic fields: [(number of asexual parasites/number of RBCs) x 100]. Subjects were seen at the time of enrollment and 30 days convalescence (post study enrollment) in which vital signs (blood pressure, heart rate, temperature and respiration) and blood was obtained. The longitudinal component of the study allowed analysis of HIV viral load trajectories during co-infection [24]. All subjects provided written informed consent in Kinyarwandan and this was recorded into the secured study documents. The study protocol, including the consent process, was reviewed and approved by the Institutional Review Board of the Albert Einstein College of Medicine and the National Ethics Committee of Rwanda. Bivariate analysis between the HIV+ and HIV- groups was conducted using the Mann-Whitney or Wilcoxon rank-sign test for continuous variables and the χ2 test for dichotomous variables. Statistical analysis was done using Graph Pad Prism (San Diego, California). A two-tailed p≤0.05 was considered statistically significant.

### Sample collection

Upon enrollment, venipuncture was performed using K_2_ EDTA blood collection tubes (BD Vacutainer, Franklin Lakes, New Jersey) of which 3mL of blood was separated to obtain plasma for antimalarial antibody measurements by protein microarray. Peripheral blood mononuclear cells (PBMCs) were collected using CPT tubes (BD Vacutainer) at study entry. PBMCs were washed with HBSS (Lonza, Walkersville, MD) and frozen at a concentration of 10^6^ cells/mL in freezing media that contained 90% Fetal Bovine Serum (Atlanta Biologicals, Lawrenceville, GA) and 10% dimethyl sulfoxide (Sigma, St. Louis, MO). Samples were placed at -80°C and frozen at -1°C/min using Nalgene Mr. Frosty freezing containers (Sigma). The samples were shipped to New York in a liquid nitrogen dry shipper. A complete cell blood count (CBC) was done for each subject at the National Reference Laboratory in Kigali, Rwanda. Plasma HIV-1 viral load assessments were performed at Montefiore Medical Center in Bronx, NY, using the Abbott Real Time HIV-1 Assay (Abbott Laboratories) with 39 copies/mL as the lower limit of detection.

### Assessing the *P*. *falciparum*-specific antibody response by protein microarray

To characterize the *P*. *falciparum*-specific antibody response we employed a microarray with 824 *P*. *falciparum* antigens (Antigen Discovery, Irvine, CA). These antigens were selected based on their consistent immunoreactivity in West Africa and other malaria-endemic regions [12, 25]. Eighteen age-matched subjects from each study group were selected for the microarray analysis. Plasma samples from the enrollment blood draw were randomly distributed across the eight microarray pads per slide. Plasma from one HIV- sample was used as an internal control on each slide. Plasma samples were diluted 1:100 in blocking buffer containing *E*. *coli* lysate and were applied to each subarray and incubated overnight at 4°C. The slides were washed and a biotin-conjugated goat anti-human IgG was added. After incubation, the slides were washed with TBS and bound antibodies were detected with a streptavidin-conjugated Cy5 fluorochrome. A final wash with TBS and water was performed in order to remove any unbound antibodies. Fluorescence intensities were measured with a Genepix 4000B Microarray Scanner (Molecular Devices, Sunnyvale, CA) at a wavelength of 635nm. The data was processed using the GenePix Pro 6.1.0.4 software and statistical analyses were done using the R Project for Statistical Computing [26].

### Phenotypic analysis of B-cell and T-cell subsets

We performed flow cytometry to identify the following cellular subsets: naïve B cells (CD19+CD10-CD21+CD27-), classical MBCs (CD19+CD10-CD21+CD27+), activated MBCs/plasmablasts (CD19+CD10-CD21-CD27+), atypical MBCs (CD19+CD10-CD21-CD27-), immature transitional B cells (CD19+CD10+) and CD4^+^ T cells (CD3+CD4+). To perform FACs analysis, frozen PBMCs were thawed in a 37°C water bath for 1 minute and complete RPMI was added. The cells were centrifuged, washed with PBS (Cellgro, Manassas, VA) and cell death was assessed using a live/dead fixable cell stain (Life Technologies). Fluorophore-conjugated monoclonal antibodies specific for the following markers were used: Brilliant Violet 421-anti CD19 (Biolegend, San Diego, CA), PerCP-Cy5.5-anti CD3, PE-Cy7-anti CD10, APC-anti CD27, PE-anti IgG, AlexaFluor 700-anti CD4 (Ebioscience, San Diego, CA) and FITC-anti CD21 (Beckman Coulter, Fullerton, CA). Each flow cytometry run had a minimum of 1x10^5^ PBMCs per sample and the FACS gating strategy for a representative sample is shown (S1 Fig). A human Fc receptor binding inhibitor (Ebioscience) was used to inhibit non-specific binding. Single-color controls, fluorescence-minus-one (FMO) controls, and compensation beads (Ebioscience) were used to ensure proper gating. Analysis was performed on a five-laser LSRII (BD Biosciences, San Jose, CA) using the FACSDiva (BD Biosciences) and FlowJo software (Tree Star, Ashland, OR). Flow cytometry data were expressed as percentages of CD19+ cells and the populations were compared between HIV+ and HIV- for each B cell subset using the Mann Whitney test.

### Protein microarray data analysis

Protein microarray data was analyzed using the R Project for Statistical Computing [26]. Intensity values were reported as Median Foreground 635nm—Background 635nm Antibody response intensities(*y*) were log2-transformed. Mean log2 NoDNA background intensity from each sample was subtracted from each target antigen intensity to remove the effects of cross-reaction. Intensity data were normalized to remove batch effects from differences among the protein array slides and their subarray pads using the robust linear model (RLM) method [27]. After transformation and normalization, we computed the correlations among the reference samples from each protein array slide and averaged all five reference samples together in order to create a single reference sample.

We separated the target probes from all of the control probes on the protein array chip and used the mean and standard deviation of the NoDNA control probes to determine which target probes were reactive. The NoDNA probes estimate background noise and cross-reaction of the antigen probes with non-specific antibodies. Any target probe with log2 intensity value greater than two standard deviations around the mean of the similarly transformed NoDNA control probes was considered to be reactive. The number of reactive probes per subject represents each subject’s antibody profile “breadth” [12], while the number of reactive subjects per antigen represents the “recognition” of that antigen. We used a negative binomial-family generalized linear model to compare the profile breadths of HIV+ and HIV- subjects. After computing breadth and recognition, the differences in the “magnitude” of the antibody responses were compared between HIV+ and HIV- subjects using an empirical Bayes moderated t-test. Venn diagrams were made using Venn Diagram Plotter to provide a correctly proportioned image [28].

## Results

### Demographic and clinical characteristics of the study cohort

To examine host responses to mild malaria with or without HIV co-infection, we enrolled 86 adults into our study of which two were excluded due to loss in follow-up. Fifty-seven subjects were HIV- and twenty-seven subjects were confirmed HIV+ (S1 Table). Subjects with HIV had a median CD4^+^ T cell count of 484 cells/μL (IQR: 303–629). Ten HIV+ subjects had viral loads that were lower than ≤ 39 copies/mL (lowest limit of detection). The remaining 17 subjects had detectable viral loads with a median of 25,641 copies/mL (IQR: 686–108,541). The majority of the HIV+ subjects were receiving cotrimoxazole prophylaxis and 17 were on combinations of NRTIs and NNRTIs (S2 Table).

Study subjects presented with symptoms including fever, myalgias, headache, joint pain and gastrointestinal symptoms. For the entire cohort, we found that the HIV- individuals were significantly younger (20 years vs 34 years, Mann Whitney, p-value = 0.022) and had significantly fewer platelets at the time of infection compared to HIV+ subjects (Mann Whitney, p-value = 0.02) (S1 Table). There were no significant differences in the number of days of illness or temperature between the study groups and there were no differences in peripheral blood parasitemia, hematocrit, numbers of white blood cells (WBCs), neutrophils, lymphocytes or monocytes. In the HIV+ subjects, we found no significant associations between *P*. *falciparum* parasitemia and CD4^+^ T cell counts.

### HIV infection is associated with reduced breadth and magnitude of *P*. *falciparum*-specific IgG responses

We employed a protein microarray with 824 *P*. *falciparum* antigens to compare IgG profiles in HIV+ (n = 18) and HIV- (n = 18) age-matched subjects plasma at the time of infection. The subjects had similar blood parasitemia, and hematologic indices (Table 1).

**Table 1:** Demographics and clinical characteristics of the HIV positive (HIV+) and HIV negative (HIV-) malaria infected patients.

Characteristic	HIV+ (n = 18)	HIV- (n = 18)	P-value
Age (years)	32 (29–37)	30 (24–37)	0.32
Sex % females (no.)	50 (9)	56 (10)	0.74
Parasitemia (%)	0.40 (0.31–0.44)	0.40 (0.40–0.50)	0.22
Temp (°C)	37.0 (36.6–37.3)	36.9 (36.1–37.7)	0.95
Days Ill	3.0 (2.0–7.0)	3.5 (2.8–6.3)	0.89
Leukocytes (x10^9^/L)	4.3 (3.0–5.9)	5.1 (3.6–5.8)	0.47
Neutrophils (x10^9^/L)	2.2 (1.3–3.4)	2.4 (1.3–3.1)	0.90
Lymphocytes (x10^9^/L)	1.5 (1.3–1.8)	1.4 (1.0–2.1)	0.83
Monocytes (x10^9^/L)	0.50 (0.34–0.59)	0.48 (0.36–0.74)	0.67
Hematocrit (%)	43 (41–46)	41 (37–44)	0.35

P-values were generated using Mann-Whitney test for continuous variables and the Chi-square test for gender. Median and interquartile values are reported.

**Fig 1:**
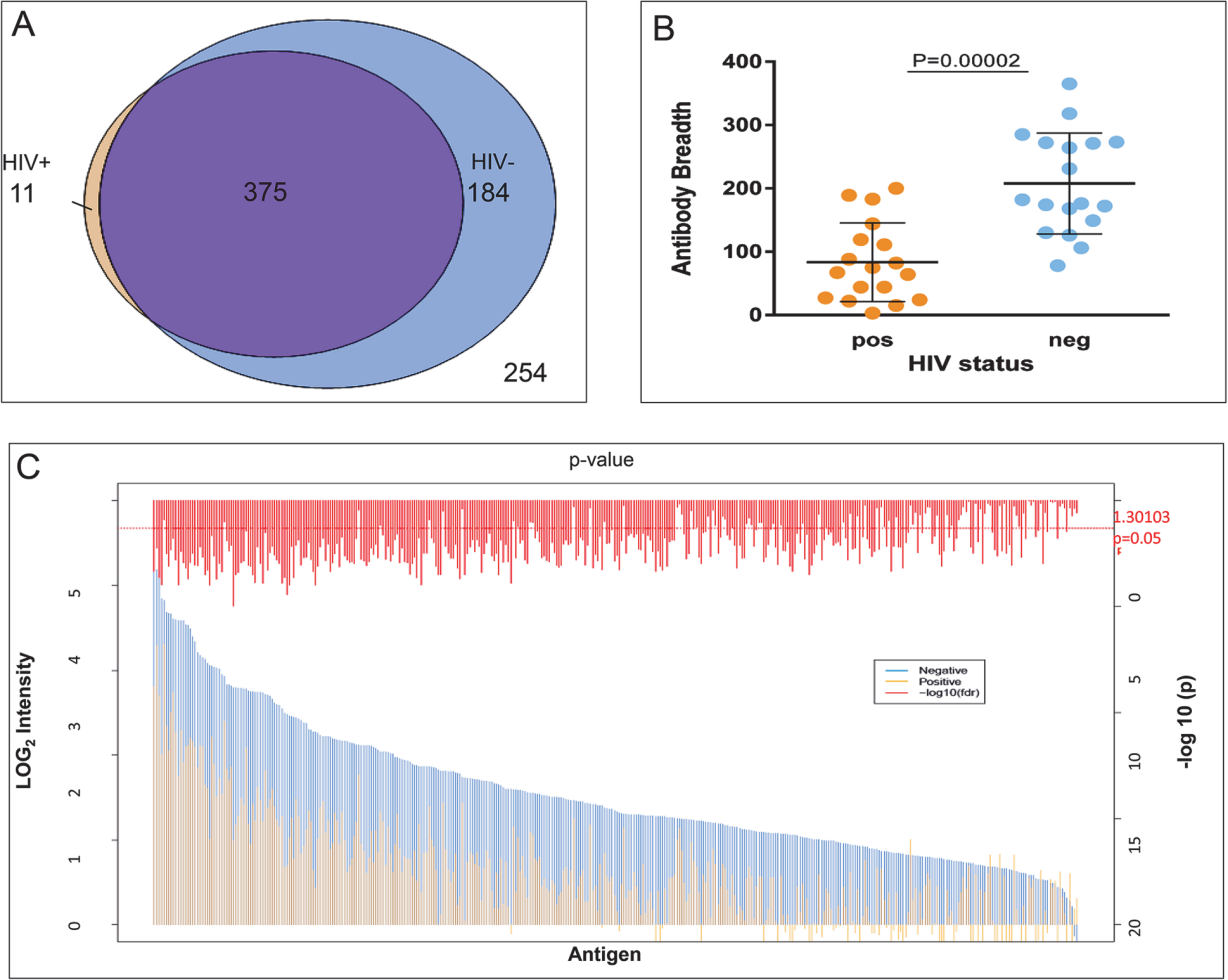
Breadth and magnitude of the IgG response to *P*. *falciparum* antigens by HIV status. (A) A microarray containing 824 *P*. *falciparum* proteins or protein fragments was probed with plasma samples from HIV+ (n = 18) and HIV- (n = 18) adults during symptomatic malaria. A. Venn diagrams showing the number of reactive antigens among HIV+ subjects (orange), HIV- subjects (blue), both HIV+ and HIV- subjects (purple) or neither (254). (B) Antibody breadth of HIV+ individuals (mean 83 antigens) and HIV- individuals (mean 208 antigens). Mean values and standard deviations are shown; Significant differences in breadth (Negative Binomial generalized linear model) (C) Magnitude of *P*. *falciparum* IgG responses by HIV status. We examined 384 antigens that were recognized in ≥ 10% of all samples and show the average IgG reactivity of each by HIV status. IgG reactivity is significantly higher in HIV- group (blue bars) compared to HIV+ group (orange bars) for 173 antigens. The red horizontal line indicates a p value of 0.05. (Empirical Bayes Moderated t-test, p<0.05, and an absolute log fold change > 1).

We defined IgG reactivity as fluorescence intensity >2 SD above the negative control (no-DNA control probe on the array). Of the 824 antigens on the array, IgG reactivity was detected against 570 (69%) in at least one of the 36 plasma samples. Among these 570 reactive antigens, IgG reactivity was detected against 375 (66%) in at least one sample of both the HIV+ and HIV- groups (Fig 1A and S2 Table). IgG reactivity against a subset of 184 antigens was only detected in samples of HIV- subjects, whereas only 11 antigens showed IgG reactivity that was exclusive to samples of HIV- subjects (Fig 1A).

Next, we compared the breadth of IgG reactivity in the HIV+ and HIV- groups. We defined breadth for each individual as the number of antigens to which IgG is reactive (>2 SD above the negative control) in a given plasma sample. We observed that the mean breadth across HIV+ individuals was lower than the mean breadth across HIV- individuals (HIV+ mean: 83 antigens; HIV- mean: 208 antigens; negative binomial generalized linear model, p = 0.00002) (Fig 1B). We then examined the overall magnitude of IgG reactivity and found it was lower in HIV+ individuals (HIV+ mean intensity: 0.14, HIV- mean intensity: 0.78; Student’s t-test, p = 0.00021). At the individual antigen level we also found that the average magnitude of IgG reactivity was consistently lower across most antigens in HIV+ versus HIV- individuals. Specifically, IgG reactivity to 173 antigens was significantly lower among HIV+ versus HIV- individuals (Fig 1C) (Empirical Bayes Moderated t-test, p<0.05, and an absolute log fold change > 1).

We then focused on the antigens to which IgG reactivity was most broadly detected across samples from HIV+ subjects and compared them to the HIV- group (Table 2). Liver Stage Antigen 3 (LSA-3) was the most broadly reactive across samples with all HIV+ and HIV- samples demonstrating IgG reactivity to this antigen. Several other vaccine candidates including: erythrocyte binding antigen 175 (EBA-175) and members of the merozoite surface antigen (MSP) family such as MSP-2 and MSP-10, were broadly reactive in both groups. Although the breadth was similar for many antigens, the magnitude of response was overall lower in HIV+ (Empirical Bayes Moderated t-test, p<0.05, and an absolute log fold change > 1).

**Table 2:** Comparison of antibody breadth and magnitude between HIV+ and HIV- samples for the P. falciparum antigens displaying the greatest breadth of antibody reactivity in HIV+ samples.

		Breadth		Differences in magnitude
Probe ID	Description	HIV+	HIV-	p.adj.eBayes**
PFB0915w-e2s1	liver stage antigen 3	100	100	0.006
PFF0995c_1o1	merozoite surface protein 10, MSP10	94	100	0.123
PFB0300c	merozoite surface protein 2 (MSP2)	89	100	0.017
PF08_0137e2s1	Plasmodium exported protein (PHISTc),	89	100	0.000
MAL13P1.176e1s2	P.falciparum reticulocyte binding protein 2	89	100	0.000
PFD1037w	transmembrane emp24 domain-containing prt	83	94	0.096
PF10_0075e1s2	asparagine-rich antigen	78	94	0.002
PFE1590w	early transcribed membrane protein	78	100	0.000
PFB0310c_1o2	merozoite surface protein 4	78	100	0.004
PF10_0025_2o2	PF70 protein	78	100	0.002
MAL7P1.176-s2	erythrocyte binding antigen 175	72	94	0.006
PF10_0356_1o2	liver stage antigen 1	72*	100	0.005
PFB0345c_2o4	cysteine protease, putative	67	94	0.001
PF08_0141e2s1	erythrocyte membrane protein 1 (PfEMP1)	67*	100	0.001
PF08_0107e2s1	erythrocyte membrane protein 1 (PfEMP1)	67	83	0.004
PFD0995ce2s1	erythrocyte membrane protein 1 (PfEMP1)	67*	100	0.022
PFB1045w	erythrocyte membrane protein 1 (PfEMP1)	67	94	0.001
PFI1475w-s1	merozoite surface protein 1, precursor	67*	100	0.003
PFB0310c-e1	merozoite surface protein 4	67	100	0.003
PF13_0197	merozoite surface protein 7 precursor, MSP7	67*	100	0.001
PF14_0495-s2	rhoptry neck protein 2 (RON2)	67	94	0.037

Frequency of detection in percent of samples by HIV status is reported. Significant differences in breadth are denoted by *, using Fisher exact test (two tailed, p value <0.05). Significant differences in antibody magnitude are denoted by **, and reported using the Empirical Bayes Moderated t-test, p<0.05, and an absolute log fold change > 1.

### Relationship between variability of *P*. *falciparum*-specific antibody responses and CD4^+^ T cell counts in HIV+ subjects

**Fig 2:**
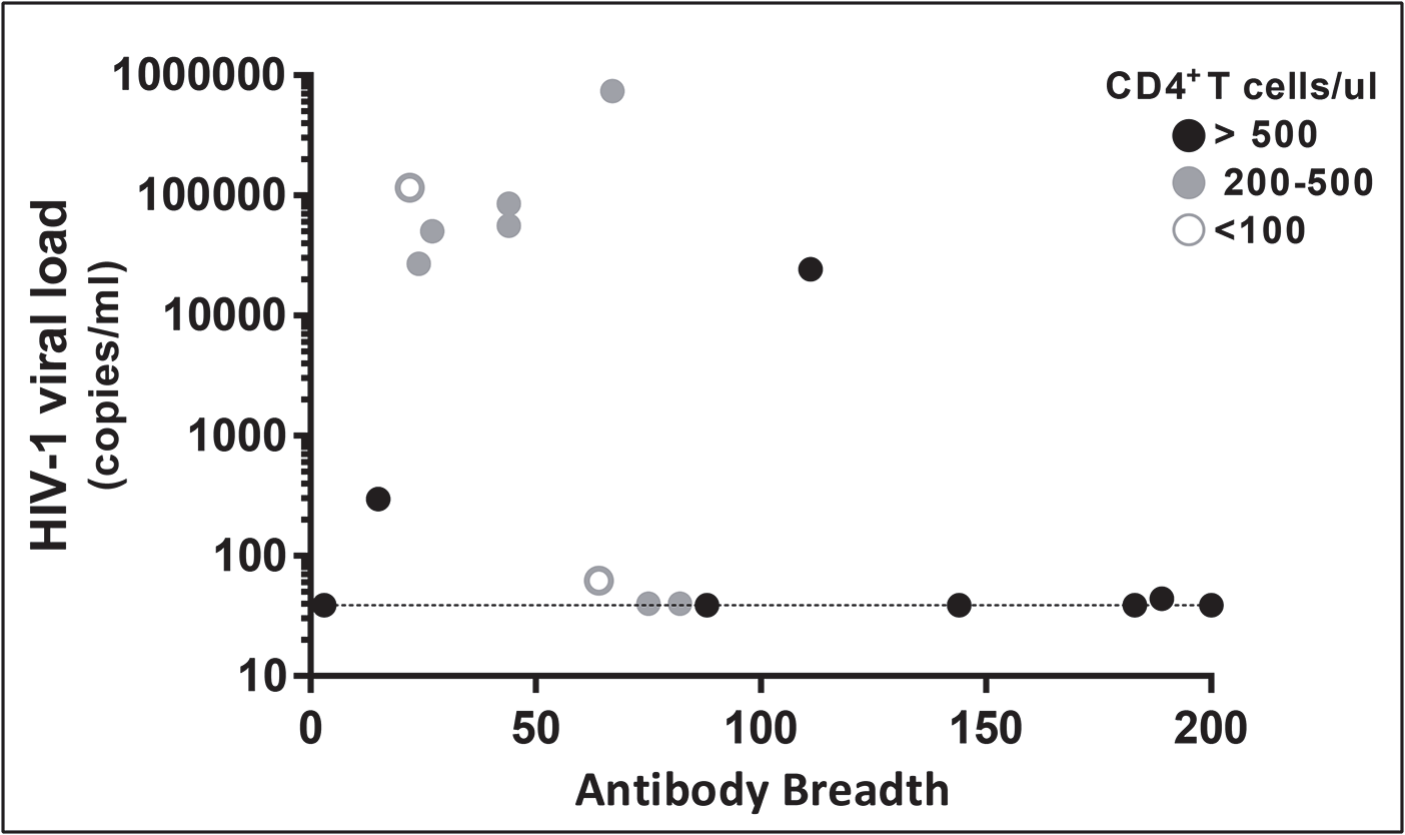
Number of reactive antibodies per sample in the HIV+ group by HIV viral load and CD4+ T cell count. The four samples with the highest antibody breadth all have CD4^+^ T cell counts >500 cells/μl and low viral loads. Dotted line denotes HIV viral load limit of detection.

We noted that HIV+ subjects demonstrated a wide breadth of antigen reactivity similar to the HIV- subjects. Thus, to explore correlates of robust antibody responses within the HIV+ group we examined the relationship between antibody breadth, absolute CD4^+^ T cell counts and HIV viral loads (Fig 2). The four HIV+ subjects with the highest antibody breadth (top 25%) had IgG reactivity to >140 *P*. *falciparum* antigens, which was similar to the antibody breadth among HIV- subjects (Mann-Whitney, p-value = 0.849). The median CD4^+^ T cell count of these same four HIV+ subjects was significantly higher than the remaining HIV+ subjects who had lower antibody breadth (617 vs 391 cells/μL, Mann-Whitney p = 0.032); and as expected, the same four HIV+ subjects had significantly lower median viral loads (39 vs 24,248 copies/mL, Mann Whitney, p = 0.03). However, high CD4^+^ T cell counts and low viral loads were not always associated with greater breadth of *P*. *falciparum*-specific antibody responses. For example, we observed three subjects who had CD4^+^ T cells >500 cells/μL and low or undetectable viral loads who had a median antimalarial IgG breadth of 15 (Fig 2). Of note, there was no difference in the reported use of cotrimoxazole or ARVs, age or parasitemia between the four HIV+ subjects with high antibody breadth and these three HIV+ subjects with low antibody breadth. Together these findings and the lack of an overall correlation between CD4^+^ T cell counts and antibody breadth in HIV+ subjects (Spearman p-value = 0.125, CI -0.13, 0.739) indicate that factors beyond CD4^+^ T cell counts underlie variability in *P*. *falciparum*-specific antibody responses in HIV infected individuals.

### HIV malaria co-infection is associated with a higher percentage of atypical MBCs compared to malaria infection alone

**Fig 3:**
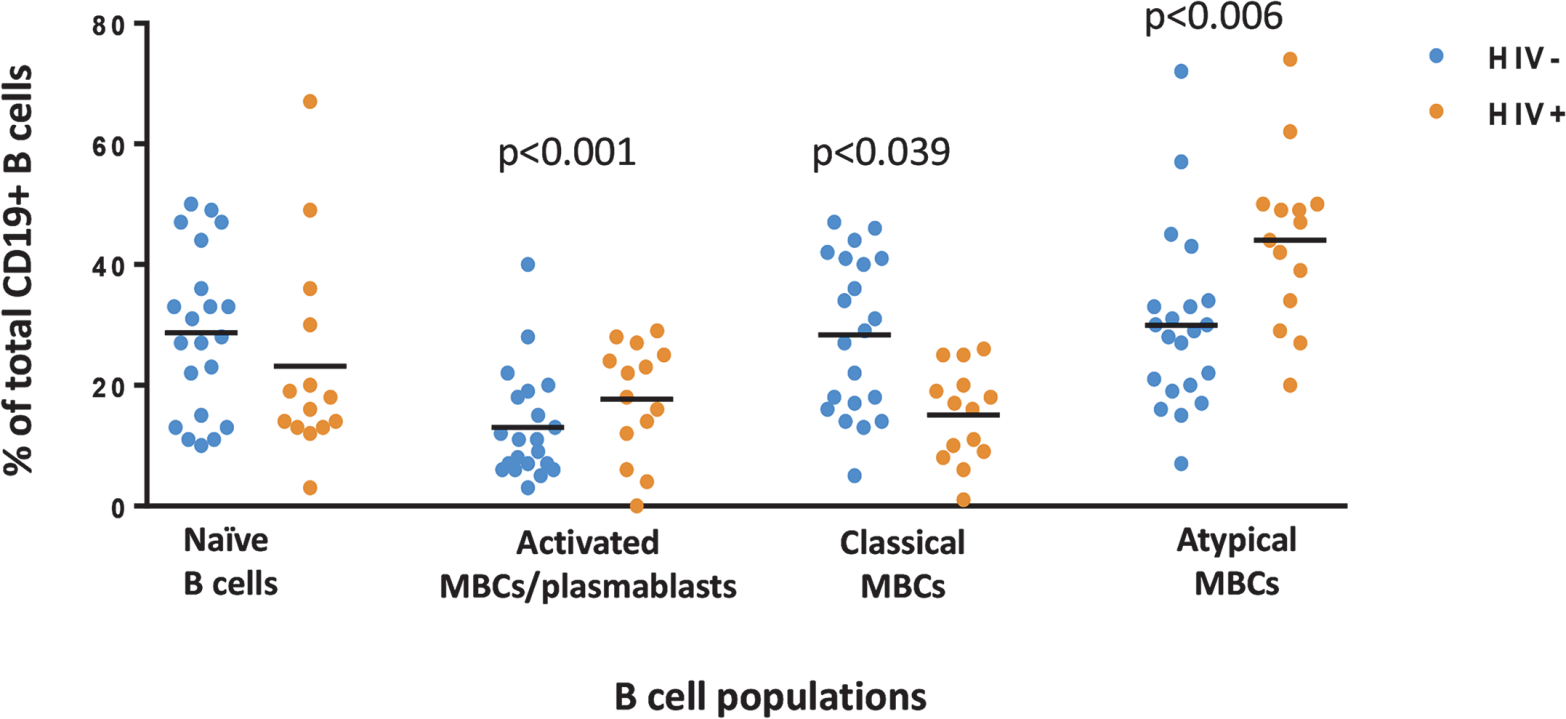
B cell subset analysis of HIV+ (n = 14) and HIV- (n = 21) subjects at the time of symptomatic malaria. The B cell subsets were determined by flow cytometry: naïve cells (CD19+CD10-CD21+CD27-), activated MBCs/plasmablasts (CD19+CD10-CD21-CD27+), classical MBCs (CD19+CD10-CD21+CD27+) and atypical MBCs (CD19+CD10-CD21-CD27-). The black bar denotes median values. The frequency was determined as percent of total CD19+ B cells. The Mann Whitney rank-sum test was used to compare variables between groups.

To gain insight into the B cell biology underlying HIV-associated reductions in *P*. *falciparum*-specific antibody responses, we evaluated B cell subsets by flow cytometry at the time of malaria infection in HIV+ (n = 14) and HIV- (n = 21) adults (S3 Table). We found no significant difference in the percent of naïve B cells between HIV+ and HIV- study subjects (Fig 3). Compared to malaria-infected HIV- subjects, the HIV+ subjects with malaria had a higher percentage of atypical MBCs (Mann Whitney, p-value = 0.0064) (Fig 3). For subjects who had both flow cytometry and protein microarray (n = 20) we observed no significant correlation between antibody breadth and the percentage of atypical MBCs. Previous studies have demonstrated that HIV or malaria infection is associated with an increase in the immature/transitional B-cell subset [29, 30], therefore we evaluated the levels of these cells in both the HIV+ and HIV- groups co-infected with malaria. We found no significant difference in the percentage of transitional B-cells between the two study groups (Mann-Whitney rank sum test, p-value = 0.8278).

## Discussion

In this study we compared antibody responses to a large panel of malaria antigens and the distribution of B cell subsets in HIV+ and HIV- malaria infected Rwandan adults. We found that both HIV+ and HIV- individuals were capable of generating IgG responses to several hundred *P*. *falciparum* antigens including the malaria vaccine candidates LSA-3, MSP-10 and MSP-2. However, the HIV+ group demonstrated a global reduction in the breadth and magnitude of *P*. *falciparum*-specific IgG responses compared to the HIV- group. We also found that HIV malaria co-infection was associated with an expansion of atypical MBCs beyond that induced by malaria alone. A subset of HIV+ individuals with high CD4^+^ T cell counts and low HIV viral loads generated antibody responses that were similar to the HIV- cohort. However, the CD4^+^ T cell count and HIV viral load only partially explained variability in the breadth of *P*. *falciparum*-specific antibody responses in HIV+ individuals.

We observed that HIV+ and HIV- subjects had similar levels of *P*. *falciparum* parasites in their peripheral blood and similar hematocrits. This is in contrast to other studies in which *P*. *falciparum*-infected HIV+ individuals tend to have higher parasite loads and a lower hematocrit compared to HIV- subjects [31–33]. The lack of difference in parasitemia and hematocrit that we observed in HIV+ and HIV- subjects in the present study may be due to the high median CD4^+^ T-cell count of 484 cells/μL in the HIV+ group, which presumably represents only a modest reduction in immune competency and is associated with less risk for recurrent malaria compared to individuals with lower CD4^+^ T cell counts [2, 34]. Unlike the HIV- subjects in this study, the HIV+ subjects had normal platelet counts during malaria infection. Thrombocytopenia is commonly associated with malaria in HIV- populations [35, 36] and our data is consistent with a recent study that also reported normal platelet counts in HIV co-infected subjects [37]. Malaria-associated thrombocytopenia has been linked to elevated levels of IgG directed to malaria antigens bound to platelets [35, 36, 38, 39]. The data here suggest that HIV infection may abrogate this phenomenon.

Past studies have shown that HIV infection is associated with lower antibody responses to a small number of *P*. *falciparum* antigens [6–8]. In an effort to explore this observation more comprehensively, we compared *P*. *falciparum*-specific antibody responses by HIV status using protein microarrays [2, 33, 40–42]. As malarial antibody responses can vary by age, transmission intensity, and time from most recent infection, we studied samples from age-matched HIV+ and HIV- individuals residing from the same region in Rwanda and whom had active infection at the time of study enrollment [43, 44].

We found that HIV- individuals reacted to a total of 559 malaria antigens, whereas samples of HIV+ individuals reacted to 386 antigens. Moreover the number of reactive antigens per sample was lower in HIV+ subjects (n = 83) compared to HIV- subjects (n = 208), demonstrating a global reduction in antibody breadth [45]. In addition to the reduction in the number of antigens to which HIV+ patients responded, we also found a reduction in the magntide of antibody responses. There were 173 antigens to which IgG reactivity was signficantly lower in the HIV+ subjects. This is consistent with observations made in HIV+ adults from Zambia where antibody levels to AMA-1 and MSP-2 were found to be significantly lower compared to HIV- individuals [46]. Multiple studies of HIV- cohorts demonstrate that antibody breadth and magnitude correlate with protection against clinical malaria [10, 11]. Thus, the decreased antibody breadth and magnitude observed in the HIV+ group may explain why this population is more susceptible to clinical malaria.

Although the magnitude of antibody response was lower in the HIV+ group, they were broadly reactive to a number of malaria vaccine candidates, including LSA-3, MSPs, and EBA-175 [47–49]. Several studies show that IgG antibodies against MSP-2 are associated with protective immunity to malaria [11, 49]. MSP-10 is one of the few antigens that resulted in equivalent breadth and magnitude between the two cohorts [50]. Data regarding immune responses to malaria vaccine candidates in both HIV+ and HIV- individuals may help inform malaria vaccine selection for regions where HIV and malaria have high endemicity.

The antibody response profiles of the HIV- subjects provide regionally matched benchmarks to interpret HIV+ responses and we identified four HIV+ samples that had antibody breadth resembling the HIV- cohort. These highly reactive samples had CD4+ T cell counts greater than 500 cells/μL and low, to undetectable HIV viral loads, and is consistent with the observation that an increase in malaria prevalence in HIV is inversely correlated to CD4+ T cell count [51]. In contrast, three subjects with similarly low viral loads and high CD4+ T cell counts had low antibody breadth. These subjects reported similar use of cotrimazole and ARVs compared to those with high antibody responses. Cotrimoxazole has been used to prevent malaria infections and some ARVs have antimalarial activity [52–55]. A reduction in malaria infections have been shown to reduce anti-malarial responses in children and the anti-malarial effect of cotrimoxazole or ARVs could be impacting the antimalarial antibody profiles shown here [12]. Our study was underpowered to determine the effect of cotrimoxazole, ARV use, or CD4+T cell nadir on antimalarial antibody profiles. Larger studies that examine the effect of these factors on antibody breadth could provide additional insights into the processes governing antibody responses in co-infection.

There are a number of mechanisms that could explain the HIV+ associated diminished antibody response, including dysregulation of B cell responses [14]. HIV infection can directly impact B cell responses to antigens, even in the presence of adequate T helper activity and prior to the decline in CD4^+^ T cells [56]. In addition, HIV infection is associated with exhausted MBCs that upregulate several inhibitory receptors and are functionally hyporesponsive [18]. Because HIV specific B cells are concentrated in this subpopulation of exhausted MBCs, they may contribute to the humoral deficiencies associated with HIV infection [57]. In the context of malaria, a phenotypically similar B cell subset has been referred to as ‘atypical’ [19] rather than exhausted because the function of these cells and whether they are beneficial or detrimental in malaria remains unclear. Yet, among all these studies none has explored these cellular subsets in the context of HIV malaria co-infections. Our data demonstrates that during malaria infection HIV+ individuals have a higher percentage of atypical MBCs compared to HIV- patients. The expansion in atypical MBCs in HIV malaria co-infected individuals may reflect an additive effect as both malaria [17, 19] and HIV [18] can cause an increase in atypical MBCs.

## Conclusions

In summary, our results demonstrate that during mild malaria in Rwandan adults, HIV infection is associated with a reduced breadth and magnitude of *P*. *falciparum*-specific IgG responses and an expansion of atypical MBCs compared to HIV- adults. Compared to prior studies, we present an analysis of antibody responses to a larger set of *P*. *falciparum* antigens during co-infection and characterize the B cell compartment, CD4^+^ T cell counts and HIV viral loads associated with antibody breadth. The absence of a strong antibody response to multiple different antigens may explain why HIV+ subjects are at greater risk for malaria. Despite this reduced response, the HIV+ group is nonetheless able to mount antibodies against malaria vaccine candidates, including LSA-3 and members of the MSP family similar in breadth to the HIV- subjects. We are also able to show that a subset of HIV+ samples are associated with a greater breadth of reactivity, though this is not completely explained by high CD4^+^ T cell counts. Future longitudinal studies are needed to address the relationship between breadth and magnitude in antibody profiles and atypical MBCs and the potential role of ARV therapies to restore normal antibody responses to malaria.

## Supporting Information

S1 Fig Phenotypic characterization of B cell subsets in a malaria HIV co-infected individual. FACS gating strategy of B cell subsets is shown using a representative PBMC sample derived from a HIV+ malaria co-infected patient.
(PPTX)

S1 Table Demographics and clinical characteristics of all the HIV positive (HIV+) and HIV negative (HIV-) subjects enrolled in the study at the time of malaria infection (n = 57). (DOC)

S2 Table Malaria antigen probe specific reactivity data for each sample (HIV+ n = 18, HIV- n = 18). Positive or negative detection of antigen by patient plasma is noted by sample and probe. HIV+ sample details include CD4+ T cell count, antiviral regiment, cotrimazole use and viral load.
(XLSX)

S3 Table Clinical and laboratory characteristics associated with HIV+ and HIV- samples that underwent B cell subset FACS analysis. (DOC)
