# Supplementary material for: HIV Malaria Co-Infection Is Associated with Atypical Memory B Cell Expansion and a Reduced Antibody Response to a Broad Array of Plasmodium falciparum Antigens in Rwandan Adults
Source: PLoS One. 2015 Apr 30;10(4):e0124412. doi: 10.1371/journal.pone.0124412 (PMC4415913; doi:10.1371/journal.pone.0124412)
Supplement: S1 Fig — FACS gating strategy of B cell subsets is shown using a representative PBMC sample derived from a HIV+ malaria co-infected patient. (PPTX) [file pone.0124412.s001.pptx]

## Slide 1
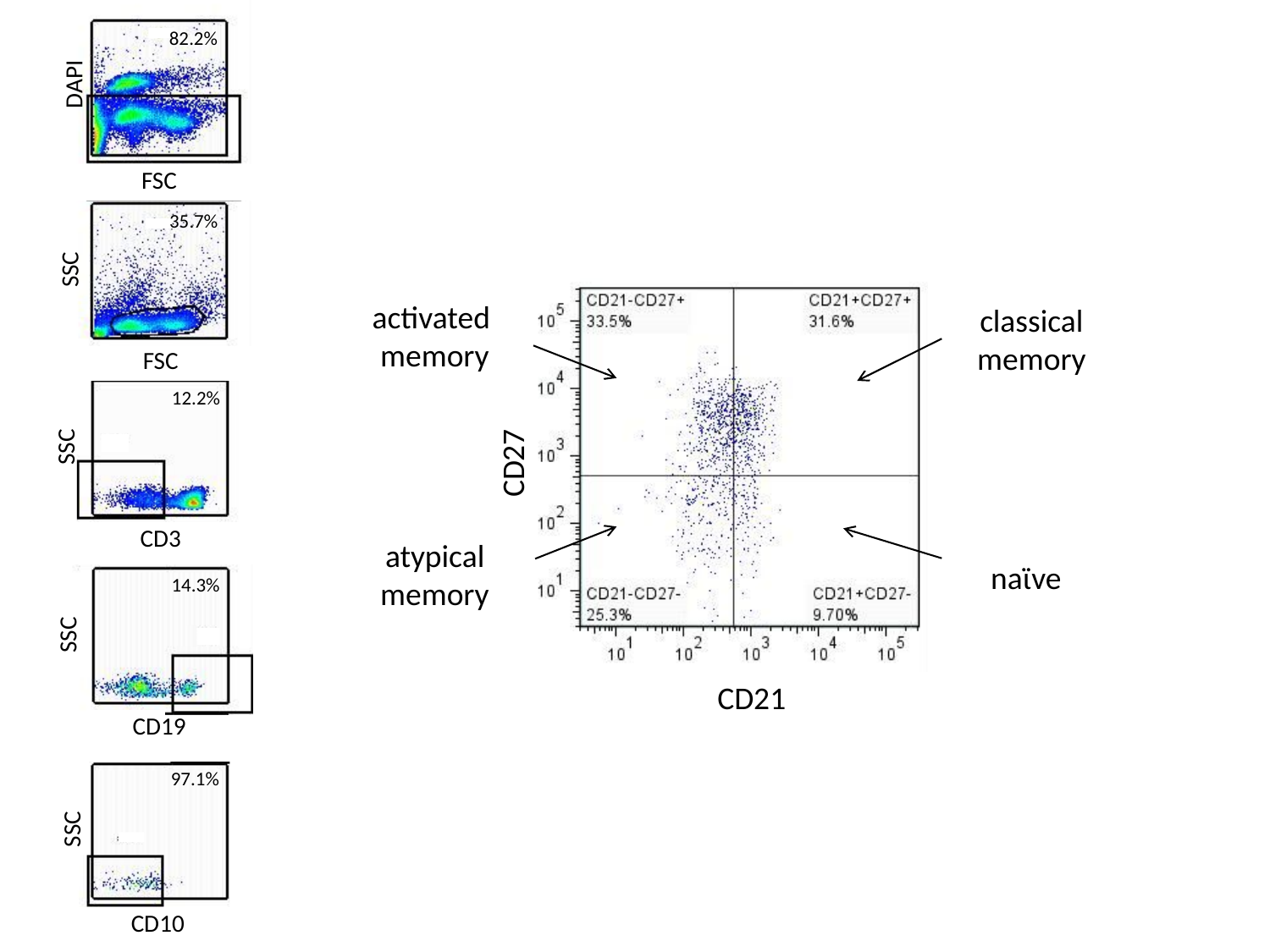

82.2%
DAPI
FSC
35.7%
SSC
activated
memory
classical
memory
FSC
12.2%
SSC
CD27
CD3
atypical
memory
naϊve
14.3%
SSC
CD21
CD19
97.1%
SSC
CD10
