# Supplementary material for: HIV Malaria Co-Infection Is Associated with Atypical Memory B Cell Expansion and a Reduced Antibody Response to a Broad Array of Plasmodium falciparum Antigens in Rwandan Adults
Source: PLoS One. 2015 Apr 30;10(4):e0124412. doi: 10.1371/journal.pone.0124412 (PMC4415913; doi:10.1371/journal.pone.0124412)
Supplement: S1 Table — (DOC) [file pone.0124412.s002.doc]

**Table S1.** **Demographics and clinical characteristics of all the HIV positive (HIV+) and HIV negative (HIV-) subjects enrolled into the study at the time of malaria infection**

P-values were generated using Mann-Whitney test for continuous variables and the chi-squared test for dichotomous variables. Median values are displayed with interquartile ranges in parentheses.
