# Supplementary material for: HIV Malaria Co-Infection Is Associated with Atypical Memory B Cell Expansion and a Reduced Antibody Response to a Broad Array of Plasmodium falciparum Antigens in Rwandan Adults
Source: PLoS One. 2015 Apr 30;10(4):e0124412. doi: 10.1371/journal.pone.0124412 (PMC4415913; doi:10.1371/journal.pone.0124412)
Supplement: S3 Table — (DOC) [file pone.0124412.s004.doc]

**Table S3. Clinical and laboratory characteristics associated with HIV+ and HIV- samples that underwent B cell subset FACS** analysis.

| **CHARACTERISTIC** | **HIV+ (n=14)** | **HIV- (n=21)** | **P-value** |
| --- | --- | --- | --- |
| Age (years) | 38.5 (31.8-40.5) | 30.0 (24.5-39.0) | 0.09 |
| Sex (% females) | 29 | 38 | 0.72 |
| Days Sick | 4 (3-7) | 3 (2-7) | 0.18 |
| Parasitemia (%) | 0.39 (0.29-0.43) | 0.50 (0.40-0.55) | 0.03 |
| Temp (°C) | 36.9 (36.5-37.2) | 36.7 (36.0-37.6) | 0.76 |
| Leukocytes (x10^9^/L) | 4.6 (3.0-6.2) | 4.6 (3.8-5.7) | 0.87 |
| Neutrophils (x10^9^/L) | 2.4 (1.9-3.5) | 3.3 (1.3-3.4) | 0.46 |
| Lymphocytes (x10^9^/L) | 1.6 (1.3-1.8) | 1.5 (1.0-1.9) | 0.73 |
| Monocytes (x10^9^/L) | 0.48 (0.30-0.70) | 0.42 (0.26-0.69) | 0.70 |
| Hematocrit (%) | 41.6 (38.8-47.2) | 41.6 (39.2-47.8) | 0.85 |
| Platelets (x10^9^/L) | 175 (146-232) | 105 (60-206) | 0.41 |

P-values were generated using Mann-Whitney test for continuous variables and the chi-squared test for dichotomous variables. Median values are displayed with interquartile ranges in parentheses.
